# Binding brain better—matching *var* genes and endothelial receptors

**DOI:** 10.15252/emmm.201810137

**Published:** 2019-02-25

**Authors:** Hannah Fleckenstein, Silvia Portugal

**Affiliations:** ^1^ Center for Infectious Diseases, Parasitology Heidelberg University Hospital Heidelberg Germany

**Keywords:** Microbiology, Virology & Host Pathogen Interaction

## Abstract

Cerebral malaria remains a major cause of death for African children, and mechanistic insights regarding the establishment of brain pathology are greatly needed. Expression of specific domains of parasite's *var* genes promoting brain adhesion of infected erythrocytes had been previously identified, but binding specificities and the receptor preference in the brain endothelial cells had not been fully described. The study by Storm *et al* (2019) in this issue of *EMBO Molecular Medicine* demonstrates that binding to brain endothelial cells via EPCR and ICAM‐1 is increased in parasites causing cerebral malaria compared to parasites causing uncomplicated malaria. Furthermore, expression levels of *var* genes encoding the CIDRα1 domain with EPCR affinity correlate with the receptor‐dependent binding to brain, but not dermal endothelial cells, highlighting the important role of EPCR in cerebral malaria pathology.

Despite a substantial decrease of the malaria burden since 2010, the latest WHO analysis indicates a stalling of the progress in recent years, in reducing both the number of malaria cases and the number of deaths caused by *Plasmodium falciparum*. Cerebral malaria is a major cause of *P. falciparum*‐caused deaths, and several mechanisms have been proposed to be involved, including obstruction of microvessels by sequestered infected red blood cells (iRBCs), endothelial dysfunction, immune activation and release of pro‐inflammatory cytokines, dysregulation of coagulation pathways, blood–brain barrier permeability or brain swelling. Although the mechanisms leading to cerebral malaria are not fully described, it appears that both parasite and host factors contribute and that iRBCs binding to the brain endothelial play an important role. The study by Storm *et al* links cerebral malaria‐causing parasites with a higher binding of iRBCs in the brain endothelium through endothelial protein C receptor (EPCR; Storm *et al*, 2019).

Remodelling of the host cell by *P. falciparum* and expression of parasite proteins on the surface of iRBCs allows its sequestration to the endothelium preventing recognition and clearance by the spleen (Smith *et al*, 1995). Adhesion of iRBCs is primarily mediated by *P. falciparum* erythrocyte membrane protein 1 (PfEMP1), encoded by the *var* genes, of which each parasite possesses ~ 60 mutually exclusive expressing genes. Binding of iRBCs can be quantified in static or flow conditions and measured by testing parasite adhesion to purified or recombinant versions of the receptors, or to primary endothelial cells from different tissues, allowing dissecting binding specificities in the former, and a better representation of the *in vivo* receptor distribution in the latter. Binding properties of different PfEMP1s have been linked to single functional cysteine‐rich adhesion domains called Duffy binding‐like (DBL) and cysteine‐rich interdomain region (CIDR). All parasites carry a similar repertoire of *var* genes, conferring each parasite a similar array of possible receptor binding phenotypes. PfEMP1s are known to bind mainly host endothelial cell receptors EPCR or CD36, via their CIDRα1 or CIDRα2‐6 domains, respectively. This phenotype dichotomy is maintained by a genetic organization of *var* genes with the subtelomeric group A *var* genes encoding EPCR‐binding PfEMP1 and telomeric group B and centromeric group C encoding CD36‐binding PfEMP1. Most parasites also carry chimeric group B/A *var* genes encoding a conserved tandem domain arrangement (domain cassette, DC) known as DC8, which also binds EPCR. Some PfEMP1 of both groups A and B bind ICAM‐1 via DBLβ domains (Lavstsen *et al*, 2003). In addition, all parasites carry one gene encoding the unusual VAR2CSA PfEMP1 binding to placental chondroitin sulphate A and expressed by parasites causing pregnancy malaria.

In years preceding the identification of EPCR as a PfEMP1 receptor, transcription of *var* genes encoding DC8/DC13 (groups B/A and A, respectively) were linked with parasites causing cerebral malaria, and parasites selected *in vitro* to bind brain endothelial cells. Lavstsen *et al* introduced for the first time a set of 42 primer pairs targeting specific CIDR and DBL subtypes and compared *var* gene transcript levels in children with uncomplicated malaria and different forms of severe malaria. The degenerate primers were designed to target semiconserved *var* sequences specific for different domain subclasses. With this tool and parasites from 88 children, the authors showed that *vars* containing DC8 and to a lesser extent DC13 domains were consistently increased in parasites from patients with severe malaria compared with uncomplicated malaria patients (Lavstsen *et al*, 2012). Avril *et al* (2012) at the same time reported that *P. falciparum* iRBCs panned on human brain microvascular endothelial cells (HBMEC) altered their binding phenotype and selected three *var* genes, including two that encoded DC8 PfEMP1. Also at the same time, Claessens *et al* analysed transcription of isogenic pairs of parasites selected on brain endothelial cells or unselected parasites with a chip containing oligonucleotide probes for multiple *var* genes from different *P. falciparum* strains. The multiple comparisons converged in outcome and indicated that on all selections, one or two *vars* showed markedly increased transcription in the brain endothelial adherent parasites and these were consistently group A *var* genes (Claessens *et al*, 2012). However, in none of the above‐mentioned studies the receptor affinity of the different PfEMP1 was determined.

It was only later that EPCR was identified by Turner *et al* as the molecular target of parasites isolated from severe malaria cases in a screening of recombinantly expressed human endothelial receptors and binding inhibition by receptor blockage. Subdomains CIDRα1.1 and CIDRα1.4 were identified to mediate EPCR binding (in DC8 and DC13 respectively), and surface plasmon resonance experiments showed that the binding occurred at significantly higher strengths as EPCRs binding to its natural ligand activated protein C (Turner *et al*, 2013). In a later study, Lau *et al* (2015) found, using structural analyses, that all but two CIDRα1 subtypes (CIDRα1.2 and CIDRα1.3 found in the *var1* pseudogenes) bind to EPCR.

More recently parasites causing cerebral malaria were shown to express *var* genes encoding for PfEMP1s that bind EPCR and ICAM‐1 in static binding assays (Tuikue Ndam *et al*, 2017). Parasites from children with cerebral or uncomplicated malaria were characterized in their transcribed *var* gene and the binding properties of iRBC collected on admission. High expression levels of PfEMP1 predicted to bind EPCR were significantly more frequent among parasites of patients with cerebral malaria than among uncomplicated malaria cases. And, when the binding capacity to CD36, ICAM‐1 or EPCR was investigated, adhesion to EPCR and ICAM‐1 was more common in cerebral malaria‐causing parasites than in uncomplicated malaria‐causing parasites. Furthermore, parasite isolates from cerebral malaria patients more frequently exhibited binding to both ICAM‐1 and EPCR, whereas binding to CD36 was more frequent in uncomplicated malaria than cerebral malaria patients (Tuikue Ndam *et al*, 2017).

However, in this study a direct link between *var* genes encoding for PfEMP1 binding to EPCR and adhesion to brain cells of cerebral malaria‐causing parasites continued to be elusive.

In this issue of *EMBO Molecular Medicine*, Storm *et al* (2019) now used a micro‐channel flow adhesion assay to enquire how inhibition of iRBC cytoadhesion through blocking ICAM‐1, CD36 or EPCR on brain or dermal endothelial cells affects binding of parasites causing cerebral malaria or uncomplicated malaria. Parasites collected from children with cerebral malaria or uncomplicated malaria were allowed to bind to HBMEC or human dermal microvascular endothelial cells (HDMEC) in flow conditions. The data show that cerebral malaria‐causing parasites bind better to HBMEC than uncomplicated malaria‐causing parasites; no such difference is observed when either type of parasite is overlaid on HDMECs. Interestingly, cerebral malaria‐causing parasites bind equally well to HBMEC and HDMEC, but uncomplicated malaria‐causing parasites adhere better to HDMEC than to HBMEC at least through ICAM‐1 binding (Fig 1). These observations are in line with earlier autopsy studies showing that CM patients have widespread parasite sequestration in most other organs apart from the brain.

**Figure 1:**
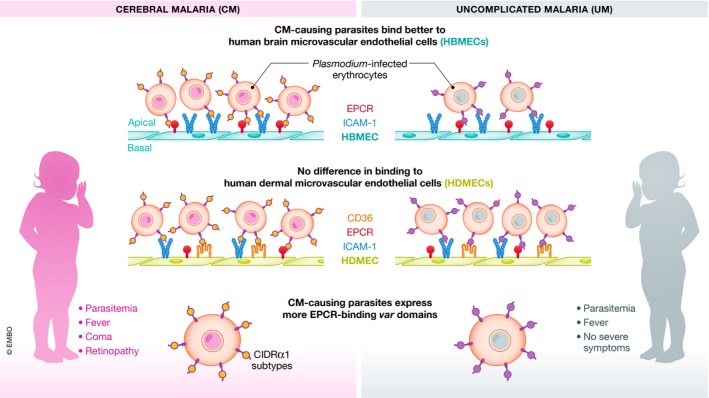
Binding properties of different *Plasmodium falciparum* clinical isolates to brain and dermal human endothelial cells Plasmodium falciparum clinical isolates from patients with uncomplicated (UM) or cerebral malaria (CM) were assessed for binding to human brain microvascular endothelial cells (HBMEC) or human dermal microvascular endothelial cells (HDMEC) in a flow assay, and associated receptors usage was analysed through receptor blocking. This simplified graph shows that higher binding to brain endothelial cells by CM‐ compared to UM causing parasites correlated with a higher expression of EPCR binding domains, e.g. subtypes of the CIDRɑ1 domain.

Storm *et al* also investigated how the blocking of different endothelial receptors would affect binding of cerebral malaria‐ or uncomplicated malaria‐causing parasites. ICAM‐1 was blocked with antibodies in both HBMEC and HDMEC, while CD36 was blocked in HDMEC, and in all conditions, antibodies could block ~ 50% of adhesion independently of the parasites originating from cerebral malaria or uncomplicated malaria cases. EPCR binding of iRBCs from cerebral malaria or uncomplicated malaria cases was reduced in HBMEC after blocking the receptor with a recombinant protein version (rEPCR), but in the dermal endothelial cells this effect was only visible for parasites originating from cerebral malaria cases. When assessing the inhibition ability of rEPCR on the different cell types and parasites origins, the authors found a trend towards a higher inhibition of binding of cerebral malaria‐causing parasites than uncomplicated malaria‐causing parasites to HBMEC, and this difference did not reach statistical significance.

In parallel, the authors characterized the *var* gene transcripts in each of the field isolates used in the above‐mentioned adhesion assay. Performing qPCR with a recently improved set of *var* typing primer pairs targeting specific CIDR and DBL subtypes normalized to a housekeeping gene, the authors calculated the relative level of *var* transcripts to the level of the housekeeping gene transcript in each sample. Interestingly, when *var* transcript levels were correlated with iRBCs binding capacities to HBMEC or HDMEC, it showed a strong positive correlation between % binding attributed to EPCR binding and expression of EPCR‐binding *var* domains by parasites from cerebral malaria patients, supporting that cerebral malaria is indeed promoted by EPCR binding of parasites to the brain endothelium.

Adding on to previous reports, Storm *et al* attempted to recapitulate in the small *in vitro* flow chambers how parasites expressing different *var* genes lead to different clinical outcomes by comparing their binding abilities to brain or dermal endothelial cells through CD36, ICAM‐1 and EPCR. Importantly, Storm *et al* use in their assays iRBCs in the 1^st^–3^rd^ cycle after blood collection expressing the native full‐length PfEMP1s, and endothelial cell lines naturally expressing their receptors, avoiding possible affinity artefacts introduced by analyses of individual recombinant PfEMP1 domains on plates coated with recombinant protein (Azasi *et al*, 2018). The data obtained are indeed supportive of EPCR being an important endothelial receptor responsible for iRBCs binding in cerebral malaria cases, alongside ICAM‐1 (Storm *et al*, 2019).

None of the main endothelial receptors to which PfEMP1s bind is selectively expressed on the brain endothelium. Thus, it is most likely that cerebral malaria‐causing parasites do not adhere specifically to the brain, but rather bind to all EPCR and ICAM‐1 expressing tissues, or even additional other receptors, such as gC1qR, which has been associated with severe malaria and rosetting, but remains to be investigated in the context of cerebral malaria (Mayor *et al*, 2011). EPCR binding in the brain endothelium by PfEMP1 subdomains may also increase brain pathology by contributing to inflammation and brain swelling. The competitive effect of PfEMP1 subdomains decreases protein C activation, possibly resulting in increased blood coagulation, inflammation, recruitment of immune cells to the endothelium and obstruction of the endothelial barrier (Turner *et al*, 2013).

And why does cerebral malaria occur only in a small proportion of the cases? Possibly because EPCR‐binding PfEMP1 is highly immunogenic and induces antibodies that cross‐react with different EPCR‐binding PfEMP1 variants (Lau *et al*, 2015). Thus, young children surviving these infections develop antibodies against EPCR‐binding subdomains early in life, in particular if they live in a high‐transmission environment as shown by Turner *et al* (2015).

Incremental knowledge of the mechanisms leading to cerebral malaria associated pathology will inform the development of much‐needed adjunctive therapies which might prevent the too many children's deaths that malaria imposes annually.
